# Chronic flooding events due to sea-level rise in French Guiana

**DOI:** 10.1038/s41598-023-48807-w

**Published:** 2023-12-07

**Authors:** Rémi Thiéblemont, Gonéri Le Cozannet, Maurizio D’Anna, Déborah Idier, Ali Belmadani, Aimée B. A. Slangen, François Longueville

**Affiliations:** 1 BRGM, 3 Av. Claude Guillemin, BP 36009, 45060 Orléans Cedex 2, France; 2 Météo France, École Nationale de La Météorologie, Toulouse, France; 3 CNRM, Université de Toulouse, Météo France, CNRS, Toulouse, France; 4 Department of Estuarine and Delta Systems, NIOZ Royal Netherlands Institute for Sea Research, Yerseke, The Netherlands

**Keywords:** Climate sciences, Natural hazards

## Abstract

As sea levels are rising, the number of chronic flooding events at high tide is increasing across the world coastlines. Yet, many events reported so far either lack observational evidence of flooding, or relate to coastal areas where ground subsidence or oceanic processes often enhance climate change-induced sea-level rise (SLR). Here we present observational and modelling evidence of high-tide flooding events that are unlikely to occur without SLR in French Guiana, where sea-level rise rates are close to the global average and where there is no significant ground subsidence. In particular, on 16 October 2020, a well-documented flooding event happened in Cayenne under calm weather conditions. Our probabilistic assessment of daily maximum water levels superimposed on SLR shows that this event can be modelled and is a consequence of SLR. As sea levels will continue to rise, we show that the number, severity and extent of such high-tide flooding events will increase across several urban areas of French Guiana, with an evolution depending on the topography. As concerns are growing regarding the economic impacts and adaptation challenges of high-tide chronic events across the world, our study provides new evidence that this early impact of SLR is emerging now.

## Introduction

Chronic flooding at high tide (hereafter chronic flooding) is an early impact of ongoing sea-level rise due to climate change^1,2^. These flooding events, also widely referred to as nuisance flooding^3–5^, occur at high tide under calm oceanic and meteorological conditions, that is, without storms, hurricanes or significant swells to raise coastal water levels above critical thresholds and inundate dry areas^6^. These events are not disasters, but can create a nuisance to people and infrastructure as they reduce the functionality of roads^7,8^ or port facilities and can negatively affect subsurface networks^9^ such as water drainage systems and resources^10^, ultimately causing sizeable economic impacts^11^ and even adversely affecting mental health negatively^12^.

Chronic flooding and its impacts have been intensively documented in the United States, where the number of flooding events at high-tide has increased rapidly and even accelerated over the recent historical period^13–17^. More frequent chronic flooding has also been attributed to relative sea-level (RSL) rise e.g., in Venice (Italy)^18–20^ and Australia^21^. Although an increase of these events in coastal region is projected globally as a consequence of sea-level rise^14,22–25^, their observed increased frequency and intensity is clearest in areas where other oceanic and geological processes take place. In particular, a major accelerator of the emergence of chronic flood events is coastal subsidence, both due to natural processes (e.g. Glacial Isostatic Adjustment^26^, hereafter GIA) and to anthropogenic activity, such as the extraction of subsurface fluids causing compaction of unconsolidated sediments^27^. For instance, the recent increase in high-tide chronic flooding along the U.S. East coast is driven by both groundwater-induced and GIA-induced subsidence^28^. Land subsidence of anthropogenic and natural origin in Venice has also been demonstrated to be a major contributor to increasing flooding occurrence^18,19^. In other areas, oceanic processes were involved: for example, the increased frequency of chronic flooding at high tide in Miami was mainly attributed to a weakening of the Gulf Stream causing an acceleration of sea-level rise^15^ and to sea-level variability due to El Niño in the Pacific^13,29^.

The fact that the majority of—but not all^21^—chronic flooding events reported today are involving multiple processes is not a surprise: we are just starting to witness the emergence of a new flooding mode caused by ongoing sea-level rise under calm weather conditions. As expected, this occurs first in areas where oceanic or geological processes enhance these events. At some point, possibly in the 2030s^24^, a rapid increase in the frequency and severity of high tide chronic flood events due to sea-level rise and tides only will take place in the US and presumably in multiple coastal regions around the world.

**Figure 1:**
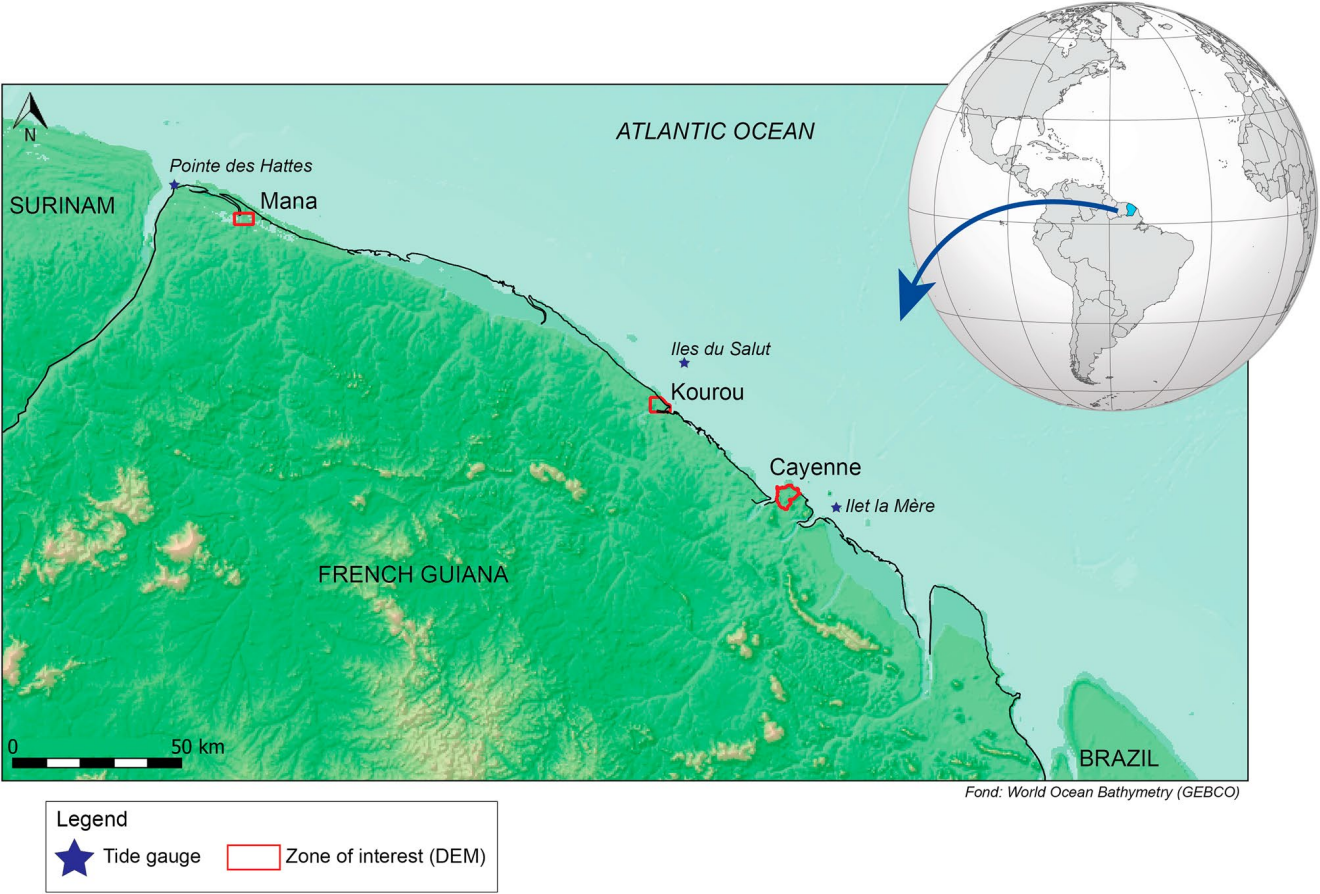
Location and map of French Guiana coastal area. The present study focuses on Cayenne and Kourou low-lying urban zones for which high-resolution Digital Elevation Model (DEM, red contour) are provided. Blue stars indicate locations of tide gauges used to characterize daily maximum water level. The map was created from QGIS Geographic Information System under the licence CC BY-SA (Open Source Geospatial Foundation Project. http://qgis.org) and the top right insert was modified from https://en.wikipedia.org/wiki/Suriname (under the licence CC BY-SA 4.0).

In this paper, we provide observational records of high-tide flooding events that are unlikely to occur without sea-level rise in French Guiana located in northern South America on the Atlantic Ocean between Brazil and Suriname (Fig. 1). RSL along the coast of French Guiana is shown to follow the global mean sea level and is not reinforced by subsidence. Flooding events are documented based on testimonials, surveys collected on the field and reports by local authorities. The physical drivers of the events are determined by combining RSL and daily maximum water levels with topography data obtained from field surveys and Digital Elevation Models (DEM). We then project the evolution of the exposure to chronic flooding in two large urban areas of French Guiana (Kourou and Cayenne) over the twenty-first century for various sea-level scenarios and a high-end sea-level scenario (“Methods”). Results not only show that the observed events are a consequence of RSL rise, but also that many areas along the French Guiana will experience chronic flooding, with important implications for coastal adaptation. Our study adds to the literature raising the urgency of adaptation to chronic flooding worldwide as more observational evidence is collected.

## Results

### Observed and projected mean sea-level change in French Guiana

In French Guiana, the longest tide gauge record is located at “Ilet La Mère” (4.89°N/52.19°W), a small island near Cayenne (Fig. 1). Despite significant data gaps in the 1980’s and around 2000, the RSL record spans more than 40 years (24 years with data), starting in 1978. The nearest Global Navigation Satellite Systems (GNSS) station, located less than 15 km away in Cayenne (4.95°N/52.31°W), shows no significant residual vertical ground motion, hence no additional vertical land motion contribution was added to the reconstructions and projections of RSL (SI Fig. 1).

**Figure 2:**
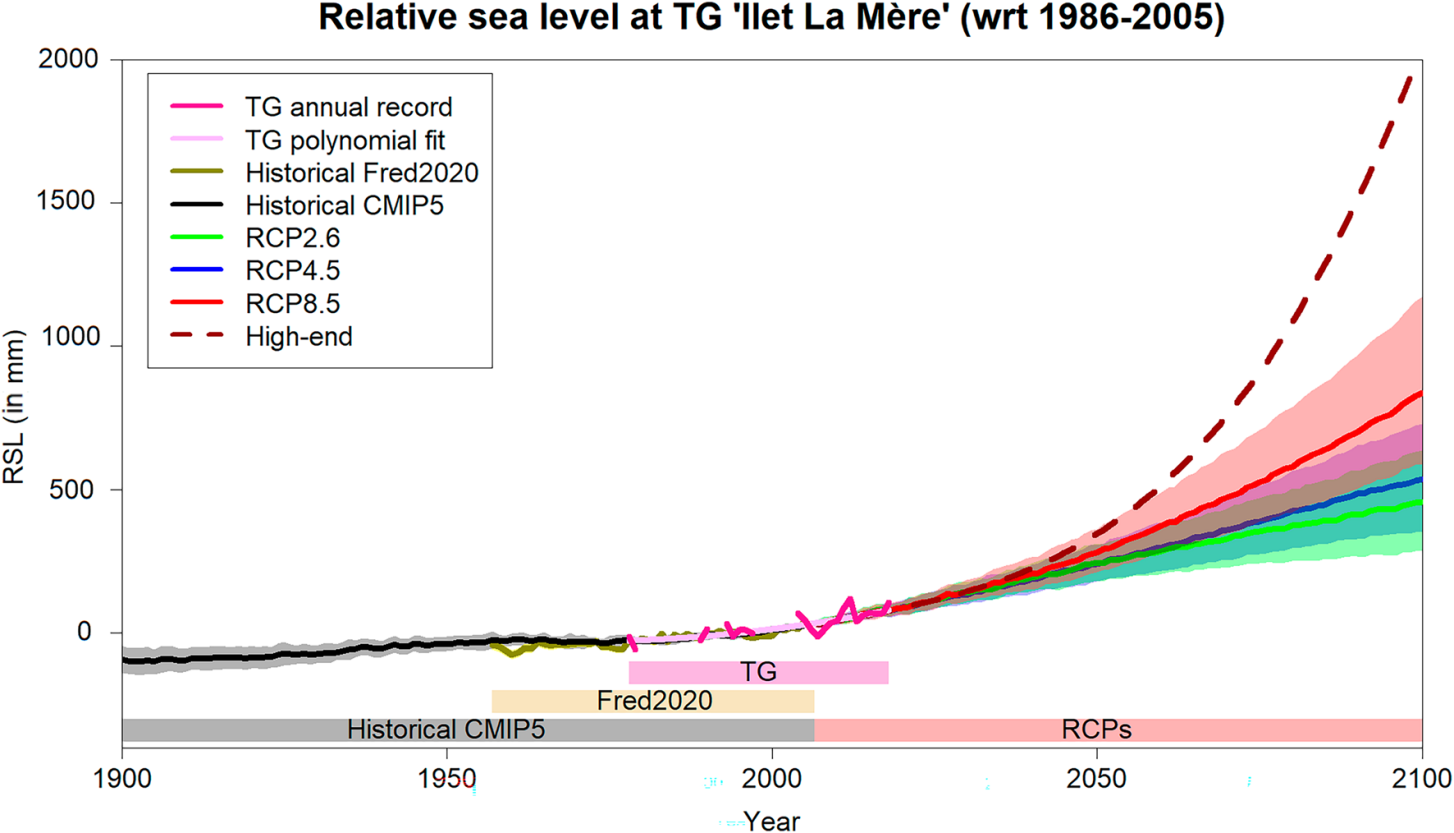
Observation, reconstruction and simulation of the annual RSL change at “Ilet La Mère” tide-gauge (TG) (4.89°N/52.19°W) over the 20th (hindcast) and 21st (projections) centuries. Projections include median (solid curve) estimates and the corresponding likely range (coloured shaded area) calculated for scenarios RCP2.6 (green), RCP4.5 (blue), RCP8.5 (red) and high-end (dashed dark red curve). Coloured bands indicate the time span of each RSL timeseries.

Over the observed period (1978–2020), the annual Coupled Model Intercomparison Project Phase 5 (CMIP5^30^) climate model-based historical reconstruction of the RSL (until 2006, see “Methods”) and its subsequent projections (after 2006, see “Methods”) compare very well with the quadratic polynomial fit of the tide-gauge record (Fig. 2). The climate model-based RSL captures the acceleration of sea-level rise. Prior to 1978, the historical model results show a slightly less but consistent sea-level rise with the regional sea-level reconstruction from Frederikse et al.^31^ (gold curve on Fig. 2). Overall, our regional sea-level hindcasts (see also “Methods”^32,33^) allows reproducing historical coastal RSL change in French Guiana. Given the good agreement between observations and model results over the historical period, the same methodological framework is used to project coastal RSL over the twenty-first century.

RSL projections at “Ilet La Mère” tide gauge for the Representative Concentration Pathways (RCPs) 2.6 (green), 4.5 (blue) and 8.5 (red) are shown in Fig. 2. For the sake of consistency with the hindcast analysis, RSL projections in this study are based on CMIP5 climate model results and SROCC glaciers, ice-sheets and groundwater estimates instead of AR6 RSL projections^34^. Until 2050, projected RSL appears to be little sensitive to the RCPs, with a median estimate (with respect to the reference period 1986–2005) varying from 24 cm (RCPs2.6 & 4.5) to 28 cm (RCP8.5) comprised within a likely range of + /- 6 to 8 cm, respectively. Beyond 2050, RSL projections start to diverge depending on the scenario, with a strong and sustained rising acceleration for the RCP8.5, yielding 84 (59–117) cm by 2100. The RSL reaches 54 (35–73) cm by 2100 for intermediate scenario (RCP4.5), while, even the most stringent scenario in terms of GHG emissions reduction (RCP2.6) still attains 46 (29–64) cm RSL rise by 2100. These estimates are very close to the global mean sea level projections from SROCC^33^. Our high-end scenario^35^ (see also “Methods”) further suggests that a RSL rise exceeding 2 m by 2100 cannot be discarded in the event of unlikely but possible ice-sheets collapse^36^.

### Analysis of historical high-tide flooding events

We interviewed local authorities from a conurbation that includes Cayenne (Communauté d'agglomération du Centre Littoral) and other cities (Iracoubo, Kourou, Ouanary) as well as port (Degrad des Cannes) officials to gather information and data on past nuisance flooding events (SI Table 1). Although several potential events were reported, most testimonies were lacking a sufficient level of detail (such as date, location, photographs, weather conditions) to perform any attribution study.

**Figure 3:**
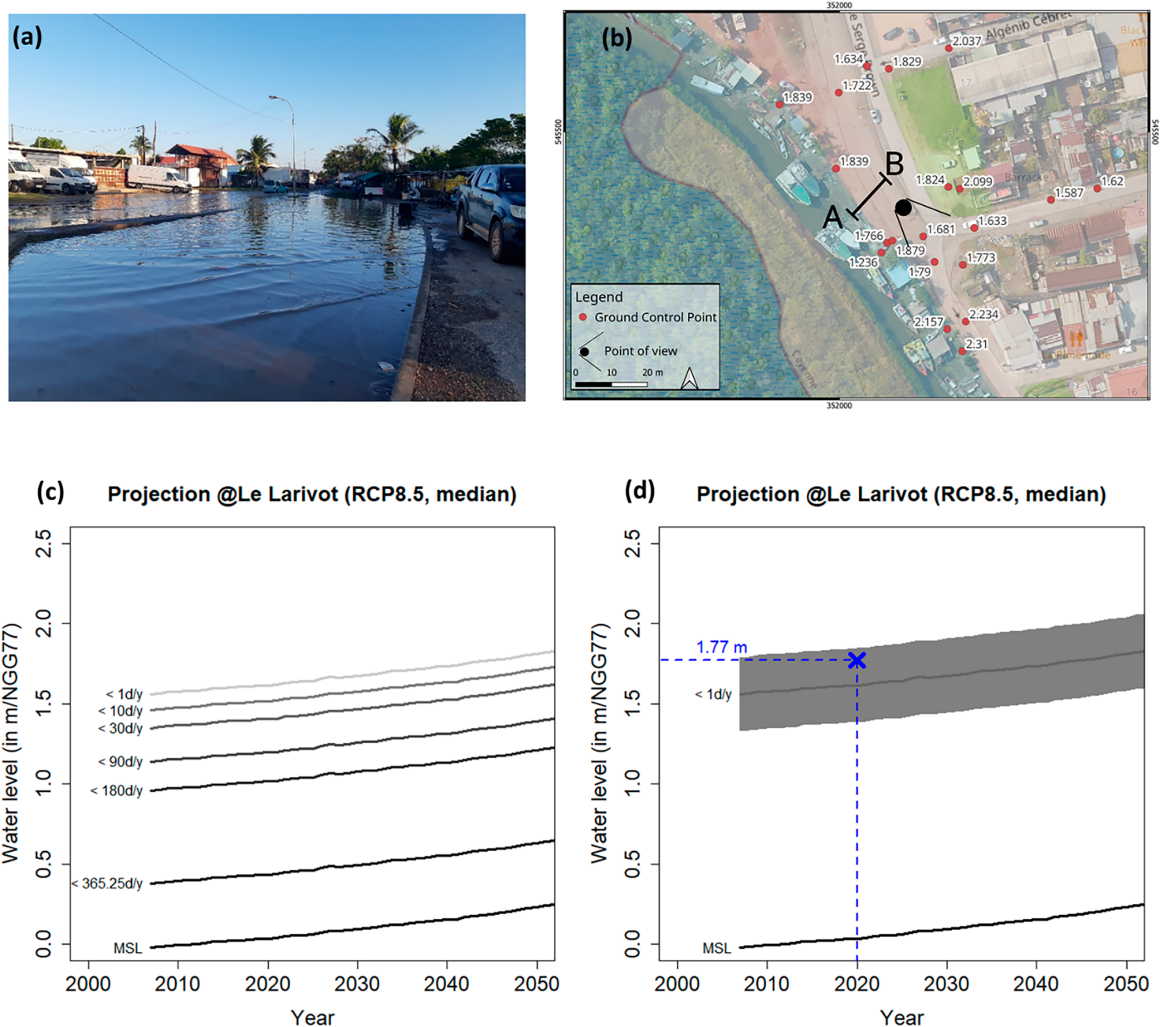
Investigation of the flooding at high tide that occurred in Cayenne on the 16th of October 2020. (**a**) Inundation photograph taken on the 16th of October 2020 at 16:00 (UTC-3) on the Serge Brown street. (**b**) Satellite image of the inundated area (in blue) together with DGPS survey point heights. The AB transect indicates where the water overflowed. The symbol with a dot and two branches indicate the point of view of (**a**). (**c**) Projected probability curves (in days/year) of daily maximum water level exceedance in Cayenne for the RCP8.5 median sea-level scenario. (**d**) Exceedance probability assessment of the 1 day/year water level in Cayenne for the RCP8.5 median sea-level scenario. Note that before ~ 2030, water level exceedance probability curves projections are insensitive to the RCP scenario. The 2-sigma uncertainty range is delimited by the grey envelope and accounts for residual surge event and DEM accuracy.

However, one event reported in Cayenne was sufficiently documented to examine its physical drivers (Fig. 3). The Serge Brown street, located in the north-east of Cayenne in the so-called “Village Chinois”, was inundated on the 16th of October 2020 (Fig. 3a). On that day, the hourly maximum water level observed by the nearest available tide gauge (Iles du Salut, 5.28°N/52.59°W, Fig. 1) was exceptionally high (exceeding 3.72 m above the hydrographic zero). Additional analysis of the tide gauge record (SI Fig. 2) shows that such a high water level has not been exceeded before 2012, while it had been exceeded 10 times between 2012 and 2020, suggesting that exceptionally high water levels can be enhanced by a rising mean sea-level. The Ile du Salut tide gauge is however located far from Cayenne (~ 50 km, Fig. 1) and local tidal amplitudes are close but not identical (SI Fig. 3). Therefore, this high frequency tide gauge record is not fully appropriate to characterize daily maximum water levels and subsequent chronic flooding events in Cayenne. While the tide gauge records near Cayenne (Larivot, Degrad Des Cannes or Ilet la Mère) would be more appropriate in principle, their frequency of measurements is not high enough and they have too large data gaps to generate reliable exceedance probability curve of water levels (“Methods”). Note also that none of the three tide gauges near Cayenne have data on the 16th of October 2020. We therefore used the predicted hourly water tidal level provided at Ilet la Mère by the SHOM to investigate whether we could reproduce the 16th October flood event at Cayenne (“Methods”).

The Digital Elevation Model (DEM) and a field survey performed with a Differential Global Positioning System (DGPS, “Methods”) revealed that the inundated area was located in a low elevation sector, i.e. < 1.70 m above the local altimetric reference (NGG77). Local testimonies also indicated that sea-water overflowed the “Leblond” channel through the AB segment, which shows a maximum elevation of 1.77 mNGG77 (Fig. 3b and SI Fig. 4). The projected evolution of daily maximum water level exceedance probability (Fig. 3c) was determined by superimposing the modelled median RSL (Fig. 2, starting in 2007) levelled within the DEM height reference system (NGG77, see “Methods”) to the probability distribution of tidal daily maximum water levels. Uncertainty sources due to residual surge events and the vertical error of the DEM were added (see “Methods”). As a result, the probabilistic assessment of daily maximum sea level shows that in 2020, the critical level 1.77 mNGG77 is within the uncertainty range of the high-tide water level exceeded one day per year (Fig. 3d). Furthermore, this critical level causing flooding at high tide in Cayenne could only start being exceeded (within the uncertainty range) since 2007 as a consequence of sea-level rise.

### Projections of low-lying zones exposed to chronic flooding

Building on the method to reconstruct past high-tide flooding events, we assess twenty-first century chronic flooding hazard evolution in two densely populated coastal cities: Cayenne and Kourou. Projections of low-lying areas exposed to chronic flooding are mapped by combining daily maximum water level exceedance thresholds from predicted tides with the topography obtained from the DEM (“Methods”, SI Fig. 5,6 and SI Table 2). In the following we describe the Cayenne case but a similar analysis for Kourou is provided in the supplementary material (SI Fig. 7).

**Figure 4:**
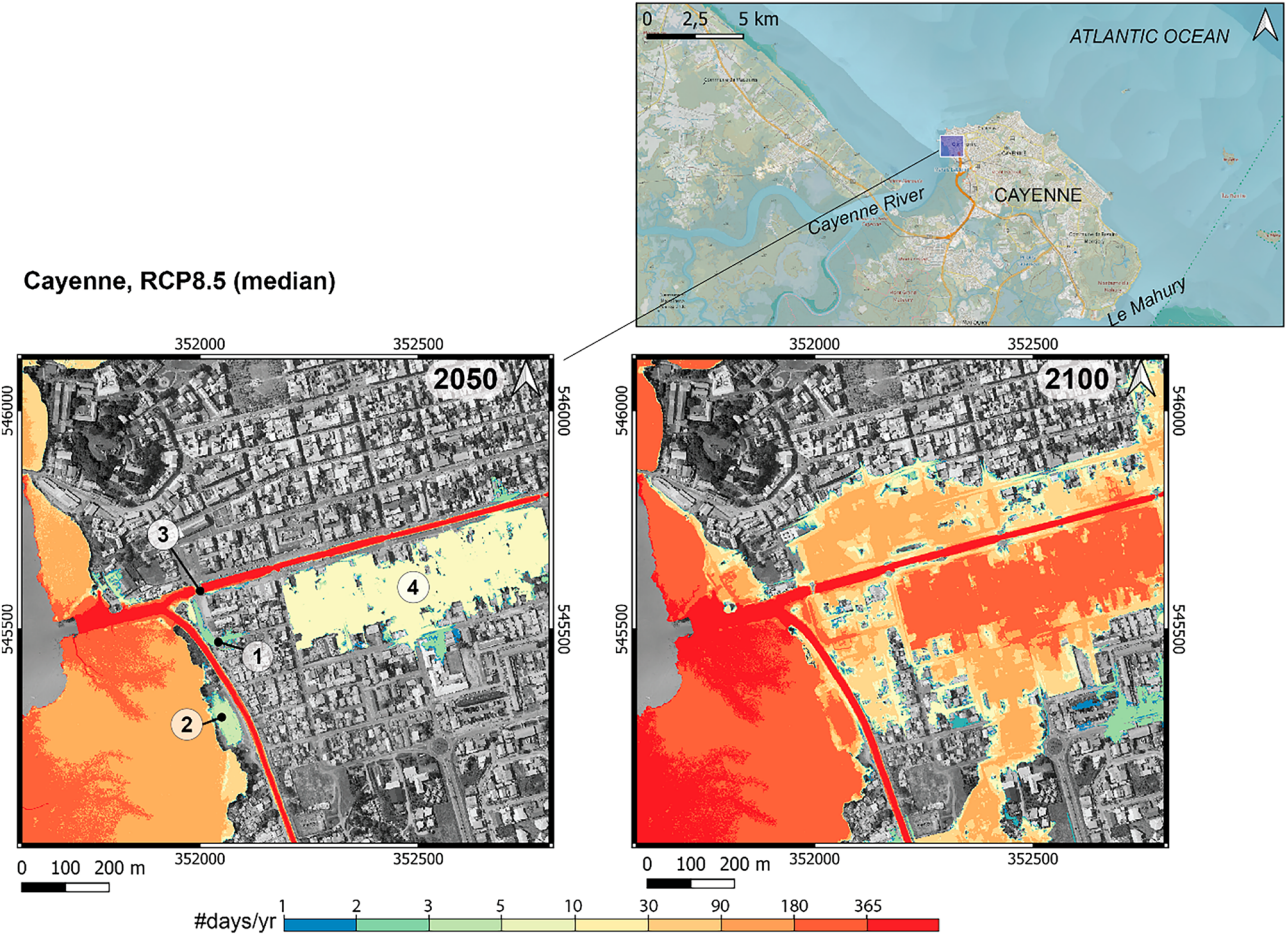
Projections of low-lying areas exposed to high-tide flooding (expressed in #days/year) in 2050 and 2100 under the RCP8.5 sea-level projections in Cayenne’s “Village Chinois” district (North-West). The mapping algorithm includes hydraulic connections at the surface of the DEM (“Methods”). Circled numbers correspond to areas that are discussed in the main text (1,2: chronic flooding recorded in the past; 3: Laussat channel sluice gate; 4: large low-lying area). Note that the largest low-lying area exposed to chronic flooding in the West/South-West is the uninhabited mangroves.

Figure 4 shows the urban zones projected to be exposed to high-tide flooding around the “Village Chinois” district of Cayenne (where the flooding event of October 2020 occurred) for the RCP8.5 scenario. Here, the mapping technique accounts for hydraulic connections at the surface of the DEM (“Methods”). As expected, the “Village Chinois” area (area #1 on Fig. 4, left) is projected to be increasingly exposed to chronic flooding in the future (5 days/year by 2050). Upstream of this area along the “Leblond” channel, area #2 also shows some exposure to chronic flooding (5 days/year by 2050). Interestingly, recent inundations were reported in this area by the conurbation, although no dates nor photographic evidence could be provided (SI Table 1, Ilet Balouin). The low-lying and expanded urban area #4 is projected to be exposed often to chronic flooding more than 10 days/year by 2050 through its hydraulic connection with the “Laussat” channel. Although the “Laussat” channel is currently closed by a sluice gate (area #3) that prevents chronic flooding of area #4, a recent maintenance check revealed some failures of the sluice gate that may affect its effectiveness^37^. We therefore considered that the sluice gate does not prevent flooding in order to raise awareness of the high exposure of the large urban zone located upstream (area #4).

By 2100, the spatial extent of urban areas exposed to high-tide flooding and the corresponding frequencies are expected to drastically increase under all scenarios, although it is largest for RCP8.5 (Fig. 4, right; SI Fig. 8). Large urban zones become connected to the sea through low-lying streets. The sluice gate of the “Laussat” channel appears to be no longer an efficient protection measure because of emerging hydraulic connections through other pathways (area #3). Although the flood-prone area and the annual occurrence are less, the same conclusion can be drawn from RCP4.5 median scenario by 2100 (SI Fig. 8).

**Figure 5:**
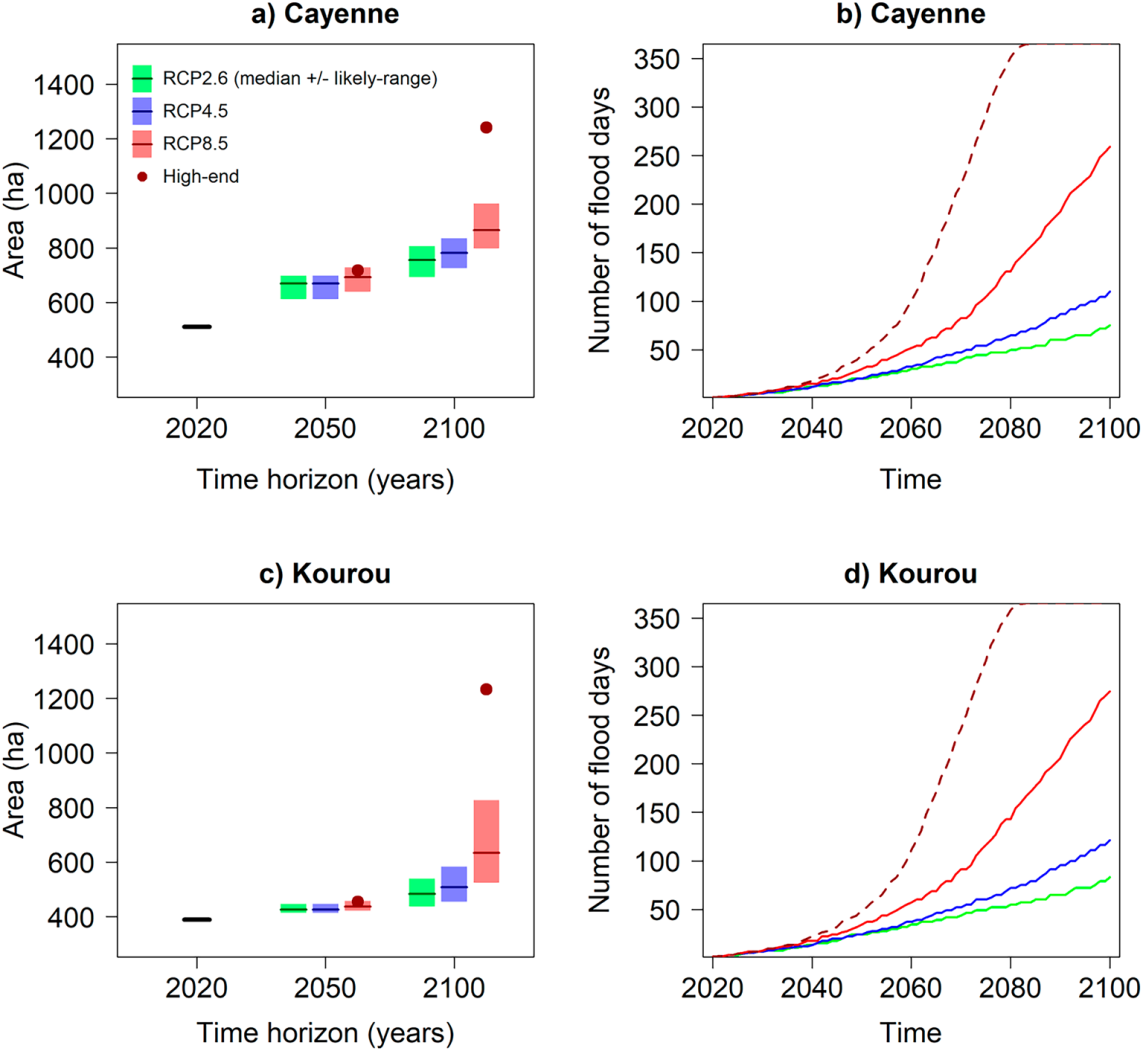
(**a,c**) Spatial extent (in ha) of low-lying areas exposure to chronic flooding at least 1 day/year in Cayenne **(a)** and Kourou **(c)** in 2050 and 2100 for the RCP2.6 (green), RCP4.5 (blue), RCP8.5 (red) and a high-end scenario (dark red dot). **(b,d)** Number of flood days (in days/year) exceeding the 1 day/year water levels of the 2020 event (used as a reference), as a function of the median RCPs and the high-end scenarios over the twenty-first century.

The two examples of Cayenne (Fig. 4, SI Fig. 8) and Kourou (SI Fig. 7) suggest that sea-level rise will trigger a substantial future expansion of low-lying urban areas exposed to chronic flooding (Fig. 5a,c) and, unless protection measures are taken, an increase in the frequency of chronic flooding (Fig. 5b,d) for the different sea-level scenarios. Both cities show distinct evolutions in terms of flooding exposure expansion. In Cayenne, the projected change of zones exposed at least 1 day/year to chronic flooding shows a significant increase of nearly 200 (± 50) ha in 2050, for a current state of 520 ha (in 2020). In comparison, the exposure increase by 2050 in Kourou is only ~ 20 ha, for a current state of 400 ha. This suggests that over the coming three decades, the city of Cayenne will face increasingly more expanded chronic flooding events, while the impact of the modest projected changes may barely be noticed in Kourou. In both cities, the chronic flooding risk frequency is however projected to strongly increase already by 2050: the 1 day/year chronic flooding water level of 2020 is to be exceeded more than 20 days/year by 2050.

In 2100, the situation differs markedly in terms of spatial exposure (Fig. 5a,c). In Cayenne, the urban area exposed to chronic flooding is projected to continue to increase but at a lesser rate than before 2050, with an increase of ~ 100 (± 60) ha between 2050 and 2100 for RCP2.6 and RCP4.5. The RCP8.5 shows a median increase of 180 ha over the second half of the twenty-first century with a slightly wider likely range than for RCP2.6 and RCP4.5. Conversely, the increase of urban area exposed to chronic flooding in Kourou accelerates between 2050 and 2100. This however strongly depends on the climate scenario considered: a median increase of ~ 50 ha is obtained for the RCP2.6 against ~ 200 ha for the RCP8.5 scenario. The likely range is also much wider and positively skewed for the RCP8.5 scenario (520–820) ha. Note that in the case of the high-end scenario, one cannot exclude a near tripling of the urban area exposed to chronic flooding by 2100 compared to present days in both cities. For both urban areas, the frequency of chronic flooding is projected to increase, but the magnitude of the increase strongly depends on the future scenario (Fig. 5b,d): our projections reveal that the annual maximum water level of year 2020 (i.e. 1 day/year level) would be exceeded ~ 2–3 months per year by 2100 under the median RCP2.6 scenario and every day by 2080 according to the high-end scenario.

Although the exposure to chronic flooding is projected to increase in terms of surface area and occurrence, our results reveal that this increase is not expected to expand at the same rate everywhere along the French Guiana coastal plain (Fig. 5). Indeed, we see that in Cayenne, the spatial exposure to chronic flooding would grow progressively but decelerate during the second half of the twenty-first century, while in Kourou, it is expected to rapidly increase after 2050, especially under a strong greenhouse gases emissions scenario. The occurrence of chronic flooding is expected to strongly increase with sea-level rise, although this increase becomes very sensitive to the emissions after 2050. These two main results should be taken into account when designing adaptation strategies, as we will discuss in the next section.

## Discussion and conclusion

### A note of caution on chronic flooding assessment and residual uncertainties

This study was motivated by a request from local authorities in French Guiana who reported several nuisance flooding events over the past recent years and were concerned by their evolution in the context of climate change. The first analyses that we conducted were thus devoted to investigating whether we could reproduce historical observations and inundation events with the models and methods that would then be employed to derive projections. This validation exercise has been overall satisfactory: first, the hindcast RSL reconstructed from climate models was well aligned with the available tide gauge records; second, we determined, from a probabilistic assessment of daily maximum water levels superimposed on RSL, that the observed high-tide flooding events could be reproduced and were a consequence of sea-level rise. Although this hindcast analysis is necessary to validate the method on which projections rely, there are several sources of uncertainty and assumptions. We discuss these in the following.

Our RSL projections (and IPCC projections in general), and more particularly the sterodynamic component^38^, are built upon a multi model ensemble mean from models that have a rather coarse resolution. This coarse resolution in turn tends to reduce internal variability in individual climate model simulation. Since the ensemble approach tends also to average out the internal climate variability, the interannual-to-decadal mean sea-level change is likely dampened in RSL projections. Recent studies have revealed that on interannual-to-decadal timescales, climate variability can significantly impact sea-level and subsequent high-tide flooding events^15,29^. Accounting for the phasing of climate variability modes in the projections (e.g. Atlantic Multidecadal Variability, Pacific Decadal Oscillation), could make the relative sea-level deviate from IPCC regional projections^39^ and would have implications for short-term to decadal predictions. A challenge for the next generation of authoritative sea-level projections will be to consider the continuity from observational sea-level records to sea-level projections and scenarios, while considering the effects of interannual variability more adequately.

Concerning the chronic flooding projections, we considered the variability of tidal amplitudes within the 18.6 years cycle, but we assume that they would remain stationary beyond this timescale. However, several studies have shown that the tidal amplitude can be significantly modulated by mean sea level change^40–42^. In addition, the French Guiana coast changes rapidly due to the landward migration of mud banks^43^, which in turn modifies the vulnerability of the coastline to erosion. Indeed, in the presence of mud banks, the incoming waves are strongly attenuated and the mud may become rapidly colonized by mangroves. As a result, the dynamics of beaches are completely muted under the protective cover of mud. Conversely, in the absence of mud, waves are no longer attenuated and the vulnerability of beaches to erosion increases^44^. Yet, such an integrated assessment is not attainable for now due to the lack of capabilities to model changing bathymetries and shoreline caused by mud banks landward migrations.

Finally, note that our two “bathtub” approaches (“Methods”) used to map low-lying areas exposed to chronic flooding likely overestimate the flood-prone areas, as they do not account for available water volume nor for the dynamics of the water flow. Nevertheless, for French Guiana coastal practitioners, our projections and maps provide a first estimate of the exposure of their territory to chronic flooding in the future. More detailed assessments would require to use advanced hydrodynamic models.

### Implications for adaptation

Coastal hazards affected or caused by sea-level rise such as chronic flooding are especially relevant in French Guiana, where the coastal plain is made of marine deposits less than 2 m above present-day mean sea level. Yet, the other dimension of coastal risks, that is, the evolving vulnerability of exposed assets, is also a significant reason of concern. As most of French Guiana is covered by a dense tropical rainforest, the vast majority of people are living in the coastal plain. The population, currently about 280,000, is rapidly growing at more than 2% per year, increasing vulnerability accordingly. Compared to other regions in France, French Guiana faces significant economic and social development challenges. Finally, besides ports and coastal cities, the region also hosts Europe’s spaceport in Kourou. In this context, coastal development in French Guiana may either facilitate coastal adaptation or make it more complex.

The increase in chronic flooding events shown in this paper could serve as an early warning for coastal managers and authorities. This will require transparent communication on the causes and consequences of these events and their projected intensification, in order to avoid responses focused on present-day or near-future hazards only. In other regions, especially in the United States, the emerging and increasing chronic flooding hazards have led to the development of near future forecasting tools for decision-support information^45,46^ as well as longer term projections^6,13,47,48^. The same could be considered here, while also considering the IPCC statement that coastal adaptation to RSL rise is more efficient when aligned with development priorities and socio-cultural values^49^.

### Implications for regional and global chronic flooding assessments

Chronic flooding events at high tide have been identified as the most urgent coastal adaptation problem because they have already started to occur^2^. Despite evidence that these events create a nuisance for people and economic activities^11^, the level of awareness about this challenge is still quite low across the world. Hence, the latest IPCC report has provided a map showing the global nature of the problem^50^ (Fig. 9.31 p. 1311). This map shows how minor extreme still water levels (defined as the 99th percentile of daily observed water levels) have been exceeded in 1995–2014 compared to 1960–1980. The map shows that these levels have been exceeded much more frequently over the recent period in the majority of areas that have experienced a RSL rise. This is consistent with the conclusions of Menéndez and Woodworth^51^ that extreme water levels in coastal areas are changing consistently with RSL changes in the majority of tide gauge locations worldwide.

To confirm that actual flooding occurrence, observations of chronic flooding events are needed. At least, land elevations above which flooding may occur should be known. This requires accurate topographic data (e.g., Lidar or GNSS surveys). In some cases, extreme water levels can be different at the tide gauge and in other nearby locations depending on currents, exposure to waves and riverine influence^52^, so that a detailed understanding of the local hydrodynamics is required as well.

Today, a national or global characterization of the chronic flooding problems is difficult due to the need for precise local observations and topographic data. Ways forward could include governments organizing surveys and reporting about chronic flooding events and the associated nuisance using their coastal observatories or citizen science as was done in Australia^21^ and the United States^13^. This is an action that could be considered in the vast majority of coastal countries and regions where sea levels are rising today.

## Methods

### Mean sea-level reconstruction and projection

For the sake of consistency, both the twentieth century reconstructions and the twenty-first century projections used in this study are based on CMIP5 climate model results. ΔRSL is extracted at the coast of French Guiana (6°N/52°E).

Over the twentieth century (before 2006), RSL simulations are taken from an ensemble of CMIP5-based historical sea-level simulations^47^, which include contributions from ocean sterodynamics, glaciers, ice sheets, groundwater extraction, reservoir impoundment, and GIA.

For the twenty-first century projections, we used results from the Special Report on the Ocean and Cryosphere in a Changing Climate (SROCC^33^) instead of more recent IPCC AR6 projections^34^. Our projections are made for Representative Concentration Pathways (RCPs^53^), based on the 21 (and 16) CMIP5 model simulations available for the RCPs4.5 and 8.5 (and RCP2.6). We discarded the MIROC-ESM and MIROC-ESM-CHEM model simulations that projected anomalously high thermosteric sea level in our study area^35,54^. For glaciers, ice-sheet and landwater components, we used the SROCC estimates. The Glacial Isostatic Adjustment contribution relies on the model of Caron et al^55^. Note that we compared our RSL projections with AR6 official products but found no significant differences between our RCP2.6 vs AR6 SSP1-2.6, RCP4.5 vs AR6 SSP2-4.5 and RCP8.5 vs AR6 SSP5-8.5 for the French Guiana coast.

To obtain the likely-range of RSL changes (*σ*_*RSL*_) within the model ensemble, we calculate the square root of the sum of the squares of each component uncertainty following the IPCC-AR5 method^56^:

1 $${\sigma }_{RSL}={\left[{\left({\sigma }_{Stero}+{\sigma }_{SMB-G}+{\sigma }_{SMB-A}\right)}^{2}+{\sigma }_{Glac}^{2}+{\sigma }_{LW}^{2}+{\sigma }_{DYN-G}^{2}+{\sigma }_{DYN-A}^{2}+{\sigma }_{GIA}^{2}\right]}^{1/2}$$

where *Stero* is the sterodynamic component; *SMB-G* and *SMB-A* are the Greenland and Antarctic ise-sheets surface mass balances, respectively; *Glac* are the glaciers; *DYN-G* and *DYN-A* are the Greenland and Antarctic ice-sheets dynamical contributions, respectively; *LW* is the landwater contribution and *GIA* is the glacial-isostatic adjustment contribution to RSL. Note that Eq. (1) assumes that contributions are independent of each other, except the sterodynamic and ice-sheets surface balance components that correlate with global-mean air temperature and which uncertainties are therefore added linearly. Assuming a complete independence between sea-level contributors tend to underestimate the uncertainty range^57^.

### High-end scenario

High-end scenarios^58–60^ explore plausible upper tail sea level scenarios beyond the likely range. They are suited for contexts when uncertainty tolerance is low and robust decision-making is preferable. There is however no unique approach to design high-end scenarios, as reflected by the recent literature that abounds in sea level projections that included high-end scenarios explicitly with various assumptions and methods^59,61–63^. Here, we rely on the high-end scenario design by Thiéblemont et al.^35^, which consists in considering the highest physics-based modelled estimate published in the literature for each sea level component. Our scenario considers that an ice-sheet collapse^36^ could happen and would lead to 2 m global mean sea-level rise in Guiana by the end of the twenty-first century.

### Vertical land motion

Local vertical land motion (*VLM*_*GNSS*_) is measured by permanent GNSS stations in Kourou (1992-onwards), Cayenne (2006–2016) and Ile Royale (2013–2022). VLM trends are provided on the SONEL portal (https://www.sonel.org) for four solutions in Kourou (University of La Rochelle ULR, Nevada Geodetic Laboratory NGL, Jet Propulsion Laboratory JPL and GeoForshungsZentrum GFZ) and two solutions in Cayenne (ULR and NGL). Residual VLMs (Δ*VLM*_*residual*_) are obtained by subtracting solid-Earth deformation land motion due to contemporary mass redistribution ($\Delta {CMR}_{SED}$) and glacial isostatic adjustment* (*$\Delta {GIA}_{SED}$*)* to the measured Δ*VLM*_*GNSS*_ over the observed period as follows:

2 $$\Delta {VLM}_{residual}=\Delta {VLM}_{GNSS}-\Delta {GIA}_{SED}-\Delta {CMR}_{SED}$$

Hence, Δ*VLM*_*residual*_ corresponds to the observed land motion anomaly that is not explained by contemporary mass redistribution and glacial isostatic adjustment. It can be used to refine relative sea-level change locally for which GIA and CMR effects are already included (and that Eq. (2) prevents to count twice)^64^.

Δ*VLM*_*residual*_ derived from GNSS stations in Cayenne (4.95°N/52.31°W, CAYN) and Kourou (5.25°N/52.81°W, KOUR) revealed no robust and statistically significant residual vertical land motion velocities (SI Fig. 1). Residual vertical land motion in Cayenne and Kourou were therefore considered as stable and were not added as contributors in RSL reconstruction and projection.

### Daily maximum water level

To account for the tidal contribution, we build a daily maximum water level distribution with respect to the local mean sea-level that is then combined with long-term changes of mean sea-level obtained as described above.

For this study, we used the predicted tides provided by SHOM (French naval hydrographic and oceanographic service) over the period 2010–2029 at tide gauges Iles du Salut (near Kourou) and Ilet La Mère (near Cayenne). These are continuous hourly predictions, based on tidal harmonic analysis of available tide gauge measurements. Note that these predictions do not include any sea-level rise trend. To generate the distribution of water levels, we first sample a full nodal cycle (i.e., 18.6 years) from the 20-year predicted timeseries, to avoid any bias in the distribution. As predicted tides are provided with respect to the hydrographic zero, the second step consists of bringing these tides back to the mean sea-level reference. After sampling the daily maximum levels from the hourly data (third step), we establish the distribution of daily maximum levels from which we finally derive the exceedance probability curve of water levels with respect to the mean sea level (SI Fig. 5).

For comparison and validation, tide gauge observations from Iles du Salut were also analysed and used to estimate the exceedance probability curve of water levels. The spring tide range at Iles du Salut is 2.25 m. The relative sea-level timeseries from Iles du Salut results from the concatenation of (i) the “Ile Royale—Anse Legoff” tide gauge hourly record from 1989 to 2008 with (ii) the neighbouring “Ile Royale” tide gauge (located a few hundred meters away) hourly record from 2006 to present. During the overlapping period (slightly longer than one year), both timeseries exhibit a coefficient of determination R^2^ of 0.998. To build the daily maximum water level, we select years that have less than 10% of missing hourly data (period 2003–2020) from the concatenated timeseries. Then, we remove the local mean sea level rise linear trend (2.87± 1.25 mm/year). As for the predicted tide timeseries, tide gauge water levels are provided with respect to the hydrographic zero: the vertical reference is accordingly adjusted with respect to the mean sea level in 2020. From this adjusted hourly water level timeseries, we select days that are fully covered (i.e., 24 h of data) and retain the daily maximum to build the distribution of daily maximum water level with respect to mean sea-level in 2020.

In contrast with the predicted tide at Iles du Salut, the hourly water level timeseries from the tide gauge and the related distribution of daily maximum water level embeds additional surge. The analysis of monthly tide gauge records revealed seasonal sea-level variations of 10 to 15 cm (not shown). The difference between observed and predicted hourly water levels over the common 2010–2020 period revealed residual surge events that do not exceed 20 cm (SI Fig. 5, 9).

### Digital elevation models

Digital elevation models (DEM) from Cayenne and Kourou were both obtained from distinct Lidar surveys in 2015. The Cayenne DEM has a horizontal resolution of 1 m, while the Kourou DEM has a horizontal resolution of 0.5 m. Both have vertical and horizontal accuracies of 10 cm. In addition, an in-situ field campaign has been performed in 2022 with a Differential Global Positioning System (DGPS) at the location of recently observed sea water inundations in Cayenne during calm weather conditions (Fig. 3, SI Fig. 6). DGPS measurements have a vertical accuracy of 5 cm. 25 control points were collected and compared to the DEM. The analysis revealed a very good agreement between in-situ observations and collocated Lidar DEM points with a coefficient of determination R^2^ of 0.97 (SI Fig. 6).

### Mapping low lying areas exposed to chronic flooding

DEM pixels are considered exposed to chronic flooding when the pixel height is lower than the daily maximum water level. Here, daily maximum water levels are converted through the exceedance probability curves (SI Fig. 5, 9 and SI Table 2) into frequency of occurrence: i.e. number of days per year where the daily maximum water level is exceeded and displayed on the maps (SI Fig. 10). Note that this method is static as it does not account for the available water volume nor for the dynamics of the water flow. It is expected to overestimate the identification of areas potentially exposed to chronic flooding, but also allows appraising the maximum extent of areas potentially exposed to chronic flooding. We also used a second mapping method, where surface DEM hydraulic connections are accounted for. In brief, for this method, a sea pixel is first flagged as “wet”, and the “wet” signal is then propagated in all directions to neighbouring pixels if their height is lower than the daily maximum water level (SI Fig. 11). Although this “bathtub” method may reduce the overestimation of the former method, one should bear in mind that it does not account for subsurface hydraulic connections. Furthermore, it still does not account for the available water volume nor for the dynamics of the water flow. This second method remains however very relevant to discard obvious low-lying disconnected areas and to analyse through which pathways a sea water inundation could occur (see e.g., maps of Cayenne, Fig. 4).

### Supplementary Information

Supplementary Information.

## Data Availability

Tide gauge records from Ile du Salut are provided by datashom at http://dx.doi.org/10.17183/REFMAR#127 (Ile Royale Anse Legoff) and http://dx.doi.org/10.17183/REFMAR#749 (Ile Royale). Tide gauge record from Ilet La Mère are also provided by datashom at https://data.shom.fr/donnees/refmar/535. Tide predictions are not publicly available but can be purchased from SHOM at https://services.data.shom.fr/support/en/services/spm. Regional mean sea-level projections produced by SROCC can be downloaded at https://www.ipcc.ch/report/ar6/sr/srocc/download/SROCC_Ch04-SM_DataFiles.zip. CMIP5 sterodynamic projections are available at https://icdc.cen.uni-hamburg.de/thredds/catalog/ftpthredds/ar5_sea_level_rise/catalog.html. Historical CMIP5 climate simulations used to construct hindcast mean sea-level can be downloaded at https://esgf-node.llnl.gov/search/cmip5/ or can be obtained upon reasonable request to the corresponding author. High-end projections are available at https://sealevelrise.brgm.fr/. GNSS vertical land motion velocities are available at https://www.sonel.org/. Basins scale sea level historical reconstructions are provided by T. Frederikse at https://doi.org/10.5281/zenodo.3862995 and GIA and GRD estimates can be downloaded at https://zenodo.org/record/3485577. The 2015 DEM of Cayenne is not publicly available but can be requested at https://geo.data.gouv.fr/fr/datasets/d6fe1545cb216b513e2c3e561f23561191540eb2. The 2015 DEM of Kourou can be accessed at https://www.geoguyane.fr/accueil.
