# Supplementary material for: Chronic flooding events due to sea-level rise in French Guiana
Source: Sci Rep. 2023 Dec 7;13:21695. doi: 10.1038/s41598-023-48807-w (PMC10709325; doi:10.1038/s41598-023-48807-w)
Supplement: Supplementary file 1 — Supplementary Information. [file 41598_2023_48807_MOESM1_ESM.docx]

**Chronic Flooding Events Due to Sea-Level Rise in French Guiana**

Rémi Thiéblemont^1,*^, Gonéri Le Cozannet^1^, Maurizio D’Anna^1^, Déborah Idier^1^, Ali Belmadani^2,3^, Aimée B.A. Slangen^4^ and François Longueville^1^.

1. BRGM, 3 av. Claude Guillemin, BP 36009, 45060 Orléans Cedex 2, France, r.thieblemont@brgm.fr.

2. Météo France, École Nationale de la Météorologie, Toulouse, France

3. CNRM, Université de Toulouse, Météo France, CNRS, Toulouse, France.

4. NIOZ Royal Netherlands Institute for Sea Research, Department of Estuarine and Delta Systems, Yerseke, The Netherlands.

*corresponding author

**Supplementary Material 1 – Analysis of vertical land motions**

| 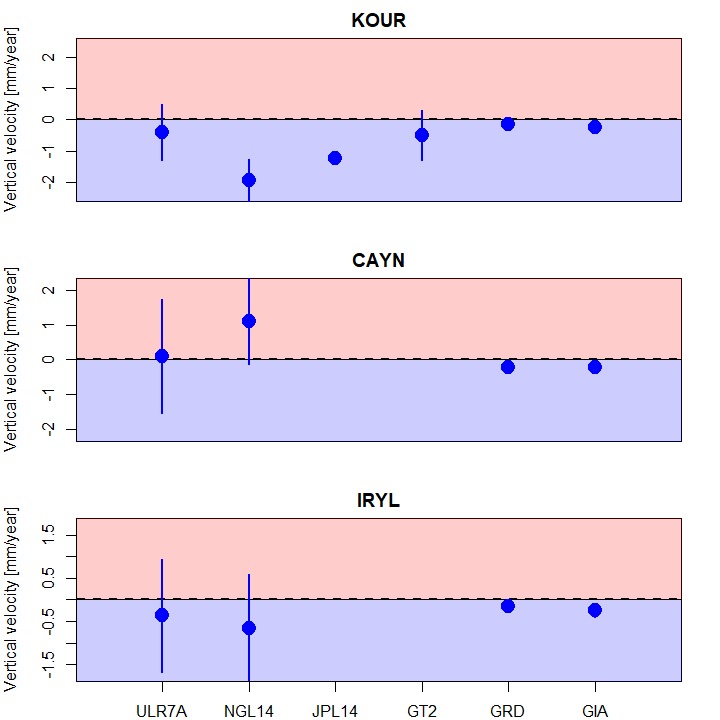 (a) | 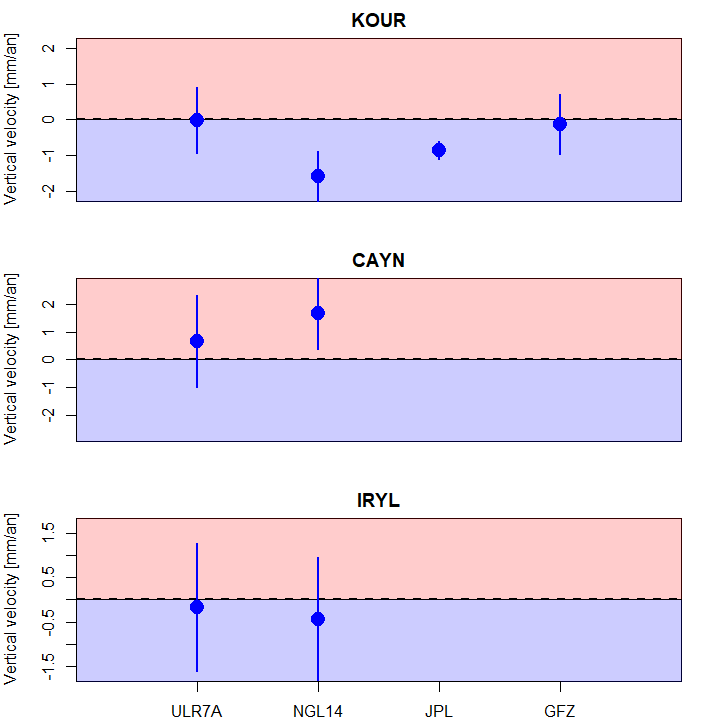 (b) |
| --- | --- |

**SI Figure 1.** (a) Vertical velocities (mm/yr) estimated at Kourou (KOUR, 52.806 °W/5.252 °N), Cayenne (CAYN, 52.310 °W/4.949 °N) and Ile du Salut (IRYL, 52.587 °W/5.285 °N) GNSS stations derived using different solutions: ULR7A, NGL14, JPL14 and GT2, and modelled for contemporary mass redistribution (GRD) and GIA. (b) Estimates of residual vertical velocities for each solution after subtracting the contemporary mass redistribution and GIA contributions. Vertical bars delimit the 1-sigma error. Out of four solutions, two indicate statistically significant local subsidence in KOUR (NGL14 and JPL14 show negative trends of -1.5 and -1 mm/yr, respectively) and two indicate no vertical velocities (ULR7A and GT2 show both null velocities).. In the absence of consensus between the four independent estimates and given the fact that the KOUR GNSS station is located well inland and far from coastal low-lying urban areas, vertical land motions in Kourou were considered as stable with no additional contribution on local relative sea-level. In Cayenne, The CAYN GNSS station results suggest uplift trends but these are not statistically significant. The IRYL results indicate no significant vertical velocities.

**Supplementary Material 2 – Synthesis of flooding events and testimonials**

Interviews were conducted with 16 public agencies and coastal stakeholders. These organizations include the Guyanese communities of cities, the coastal cities of French Guyana, the state agency and the anglers of the “Village Chinois” in Cayenne. Testimonials were collected either by email, telephone or in person. Interviews followed the same protocol: we first described what defines a chronic flooding and then asked always the same question: have you had knowledge of chronic flooding incident on your territory? If yes, is it documented (location, date, time, photo)?

**SI Table 1**. Summary of some flooding events reported on the French Guyana coastal plain territory.

| City | Date | Source | Photo | Location | Level of confidence (1 low to 5 high) | Comments |
| --- | --- | --- | --- | --- | --- | --- |
| Kourou | Unreported | City Hall (Malaganne) | No | Avenue de l'anse  (5.170N/  52.642W) | 3 | Inundation at high tide through water pipes |
| Kourou | Unreported | City Hall (Malaganne) | No | village Saramaca  (5.158N/  52.637W) | 3 | Inundation within the center of  Village Saramaca |
| Ouanary | Unreported | City Hall | No | Airfield runaway  (4.211N/  51.667W) | 2 | Partial inundation at every high tide |
| Remire-Montjoly | 24/06/2021 | Harbour authority | No | Degrad des cannes harbour  (4.854N/  52.274W) | 2 | Inundation during spring tides – flooding potentially already observed in April 2021 |
| Cayenne | 16/10/2020 | Communauté d'agglomération du Centre Littoral (CACL) | Yes | Rue Serge Brown  (4.934N/  52.335W) | 5 | Inundation during spring tides |
| Cayenne | Unreported | CACL | No | Ilet Balouin  (4.932N/  52.334W) | 4 | Near channel houses |
| Iracoubo | Unreported | Department of General Services | No | Place Ulrich Sophie | 4 | Inundation at every high tide |

**Supplementary Material 3 – Supplementary analysis**


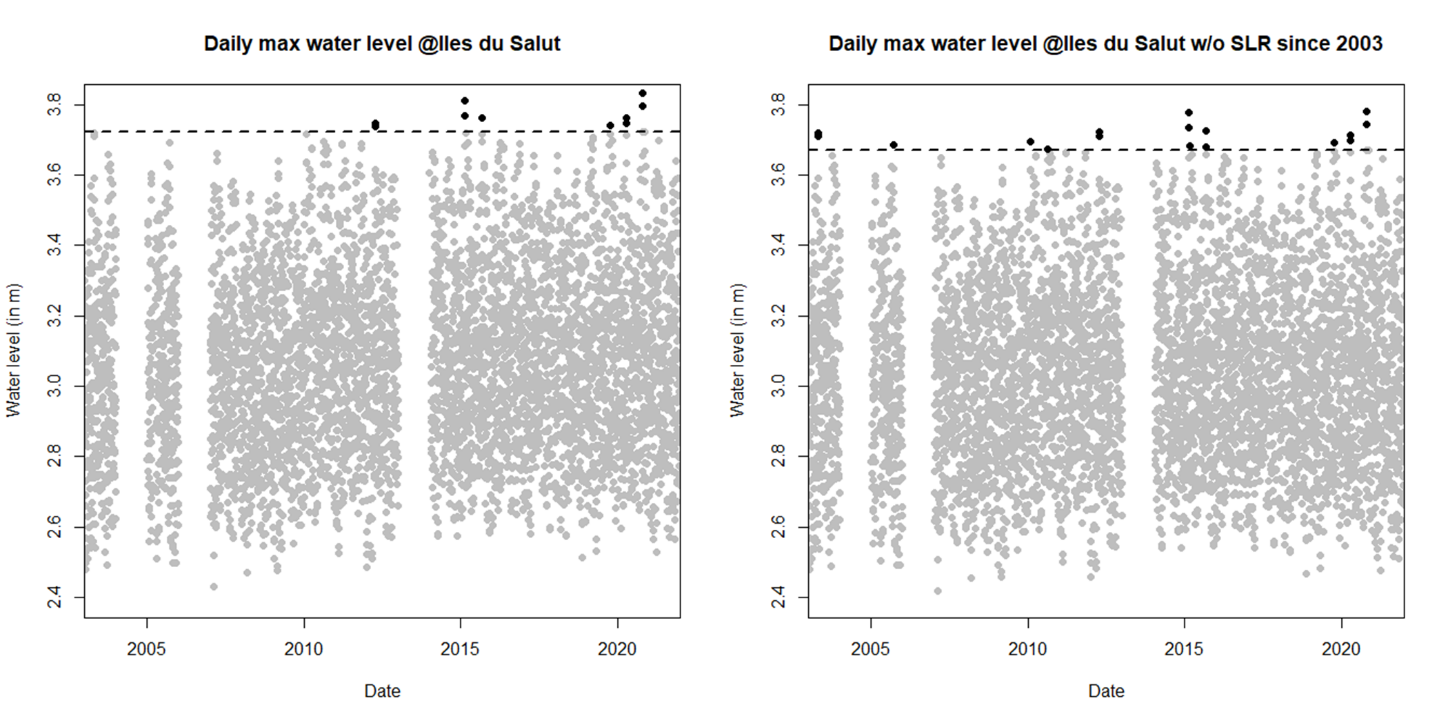


**SI Figure 2**. Daily maximum water levels measured at Iles du Salut tide gauge (with respect to the hydrographic zero reference level) before (left) and after (right) removal of the mean sea-level trend measured by altimetry (2.87 mm/year). The trend removal leads to a deviation of ~5 cm in sea level between 2003 and 2021. Black dots indicate days when the critical level which led to the flooding of 16 October 2020 (horizontal dashed line) is exceeded. This analysis suggests that such a high water level can be exceeded very rarely, and starting from 2012 because of SLR (i.e. 10 times over the whole period). In the absence of SLR over the period recorded by the tide gauge, the water level on that day would still be exceptionally high but could have been exceeded several times before 2012.

**Supplementary Material 4 – Tide spatial variability**


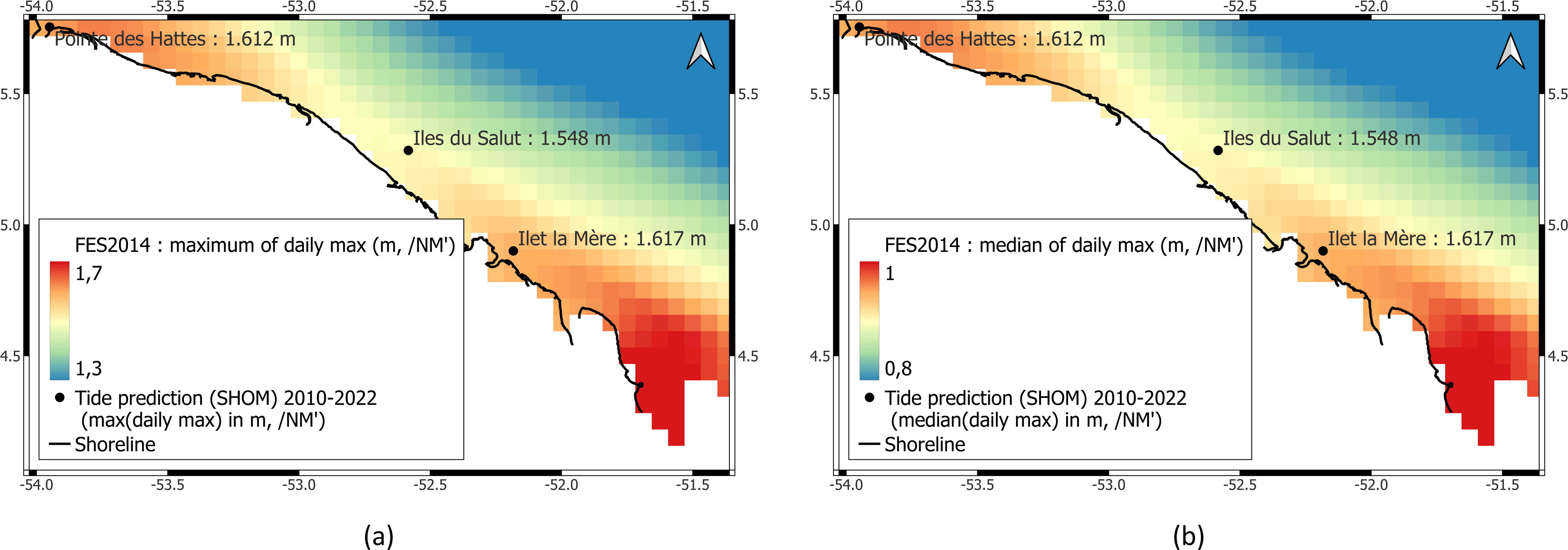


**SI Figure 3.** (a) Highest astronomical tides and (b) median of daily maximum levels with respect to the mean sea level derived from the Finite Element Solution (FES2014, Lyard et al. 2021) tide model (shading) and from the tide gauges where predicted tides (derived from observations and provided by SHOM) were used (black dots, Supplementary Material 9).

**Supplementary Material 5 – Tide spatial variability**





**SI Figure 4.** Height profile associated with the AB transect shown on Figure 3b. The maximum height that water overtopped to inundate the Serge Brown street was 1.77 mNGG77.

**Supplementary Material 6 – Daily maximum water level distribution from predicted tides**


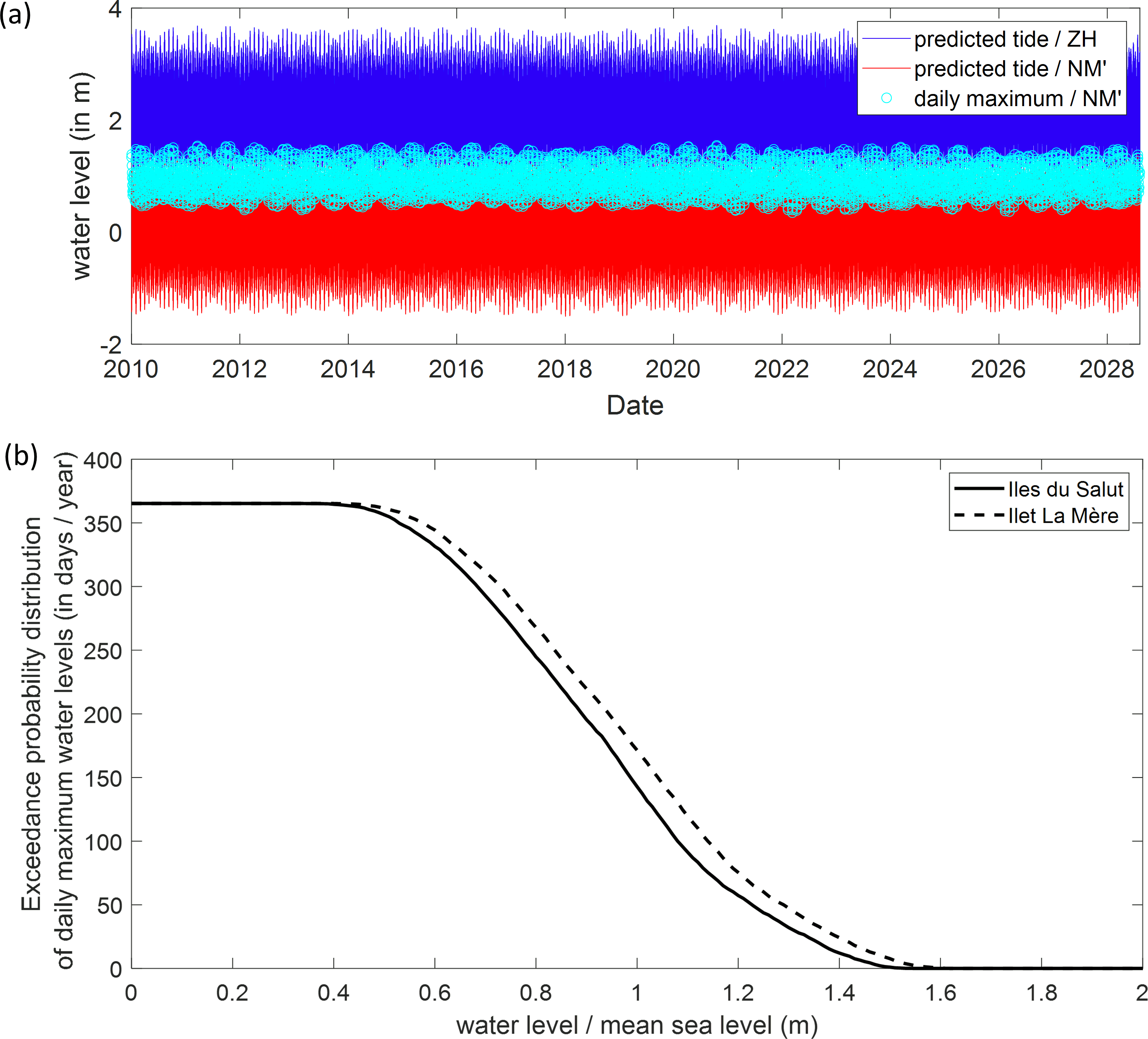


**SI Figure 5**. Processing of predicted tide timeseries provided by SHOM. (a) Hourly predicted tides at “Iles du Salut” tide gauge over the period 2010-2029 with respect to the hydrographic zero datum (blue) and to the mean sea level (red). Daily maximum water levels due to tides are selected (cyan) to construct the (b) exceedance probability distribution (in days/year) of daily maximum water levels. Exceedance probability distribution (in days/year) of daily maximum water levels is also shown for Ilet La Mère tide gauge (dotted curve) using the same method. The difference between the curves from the two tide gauges indicates that tidal amplitude differs between these two locations (larger at Ilet La Mère) and needs, therefore, to be accounted for in the chronic flooding assessment.

**Supplementary Material 7 – Daily maximum water level projections**

**SI Table 2**. Daily maximum water levels (cm) projected to be exceeded 1, 10 and 100 days/year in 2050 and 2100 as a function of Representative Concentration Pathways (RCPs). Median (bold) and likely-range (brackets) estimates are provided.

| Cayenne / Larivot (in cm) | | RCP2.6 | RCP4.5 | RCP8.5 | High-end |
| --- | --- | --- | --- | --- | --- |
| 2050 | 1d/yr | **177** (171-183) | **177** (171-183) | **181** (174-189) | 187 |
|  | 10d/yr | **167** (161-173) | **167** (161-173) | **171** (164-179) | 177 |
|  | 100d/yr | **133** (127-139) | **133** (127-139) | **137** (130-145) | 143 |
| 2100 | 1d/yr | **198** (181-216) | **206** (188-226) | **236** (212-270) | 358 |
|  | 10d/yr | **188** (171-206) | **196** (178-216) | **226** (202-260) | 348 |
|  | 100d/yr | **154** (137-172) | **162** (144-182) | **192** (168-226) | 314 |

| Kourou (in cm) | | RCP2.6 | RCP4.5 | RCP8.5 | High-end |
| --- | --- | --- | --- | --- | --- |
| 2050 | 1d/yr | **183** (177-189) | **183** (177-189) | **187** (180-195) | 196 |
|  | 10d/yr | **175** (169-181) | **175** (169-181) | **179** (172-187) | 185 |
|  | 100d/yr | **141** (135-147) | **141** (135-147) | **145** (138-153) | 151 |
| 2100 | 1d/yr | **204** (187-222) | **212** (194-232) | **242** (218-276) | 364 |
|  | 10d/yr | **196** (179-214) | **204** (186-224) | **234** (210-268) | 356 |
|  | 100d/yr | **162** (145-180) | **170** (152-190) | **201** (176-234) | 322 |

**Supplementary Material 8 – Cayenne topographic survey**


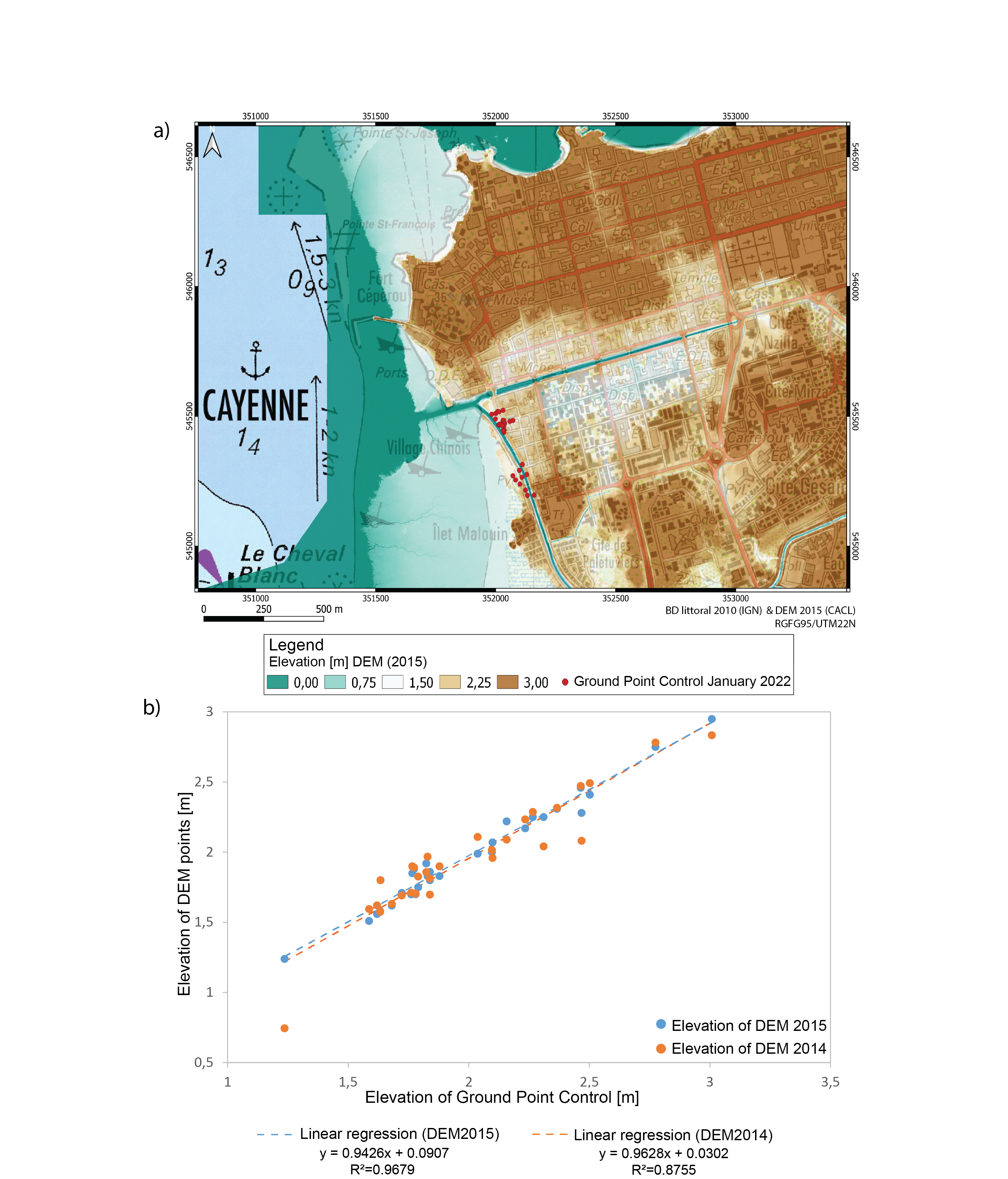


**SI Figure 6.** a) The Digital Elevation Model (DEM) for Cayenne (2015) used in our study and field topography survey in 2022 (with DGPS, red dots). b) Scatterplot between DGPS survey heights (2022) and Lidar DEM of 2014 (orange) and 2015 (blue). Vertical reference system is NGG77. DEM2015 shows a better agreement with the DGPS survey than DEM2014. Vertical standard error of DEMs are 10 cm.

**Supplementary Material 9 – Areas exposed to chronic flooding in Kourou**

SI Figure 7 below shows a zoom of low-lying areas projected to be exposed to high-tide flooding around the district of “village Saramaca” for the RCP8.5 scenario. This area was recently exposed to such flooding (Supplementary Material 2), but unlike the October 2020 event in Cayenne, no precise dates or photos could be collected. Our results indeed reveal a low-lying urban area centered on “village Saramaca” that is exposed to high-tide flooding (location 1) by 2050, which strongly expands by 2100 under the RCP8.5 median scenario. Note that the exposed urban area denoted 1 on the 2050 map is not hydraulically connected to the sea through the channels. This exposed area could be identified only by using the static method to map low-lying areas (see Methods).


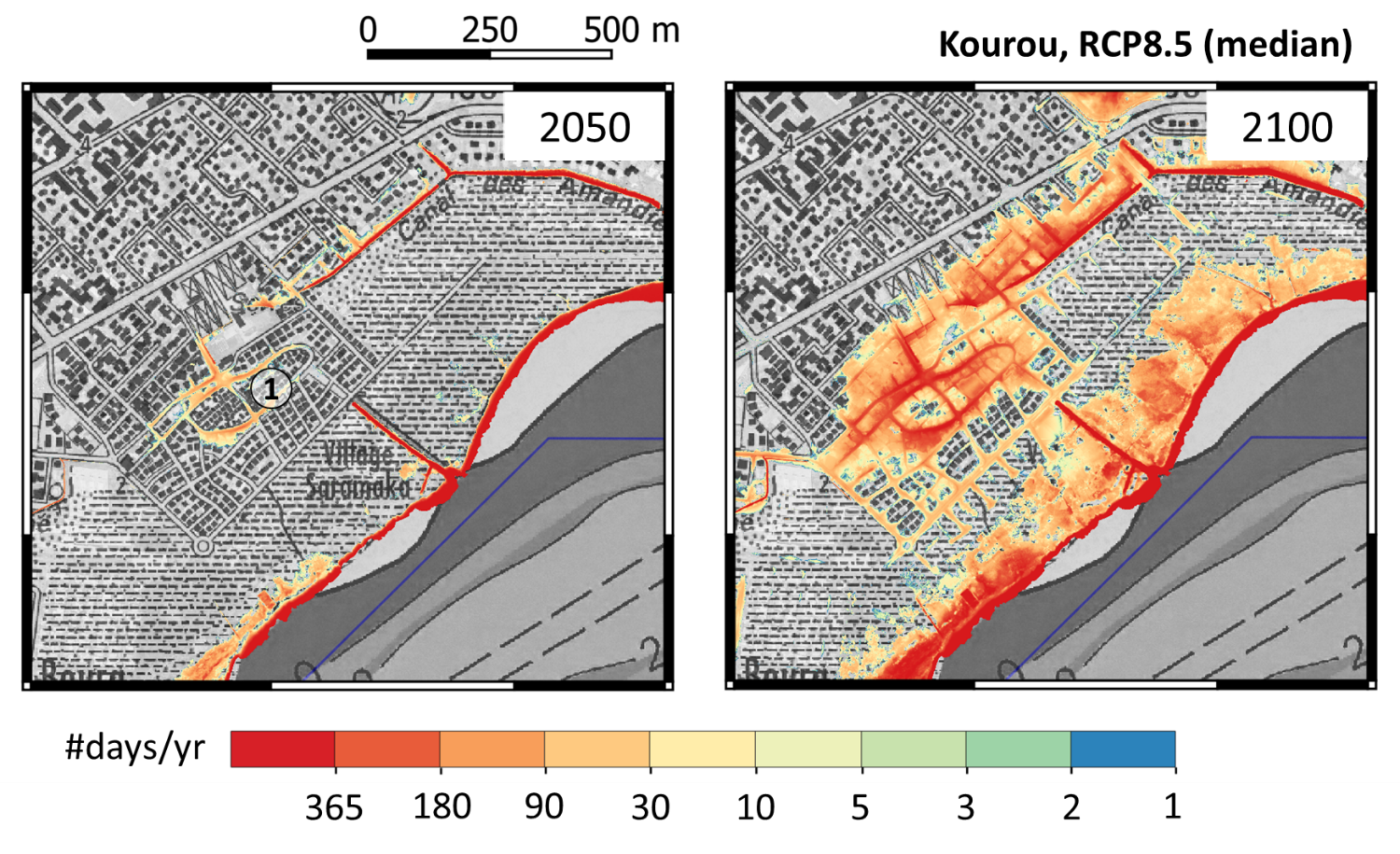


**SI Figure 7.** Projection of low-lying areas exposed to high-tide flooding (in days/year) in 2050 and 2100 for the RCP8.5 scenario in Kourou’s “Village Saramaca” district (South). Circled number 1 corresponds to the area of nuisance flooding recorded in the past (“village Saramaca”). The mapping algorithm does not include hydraulic connections at the surface of the DEM.

**Supplementary Material 10 – Areas exposed to chronic flooding in Cayenne**


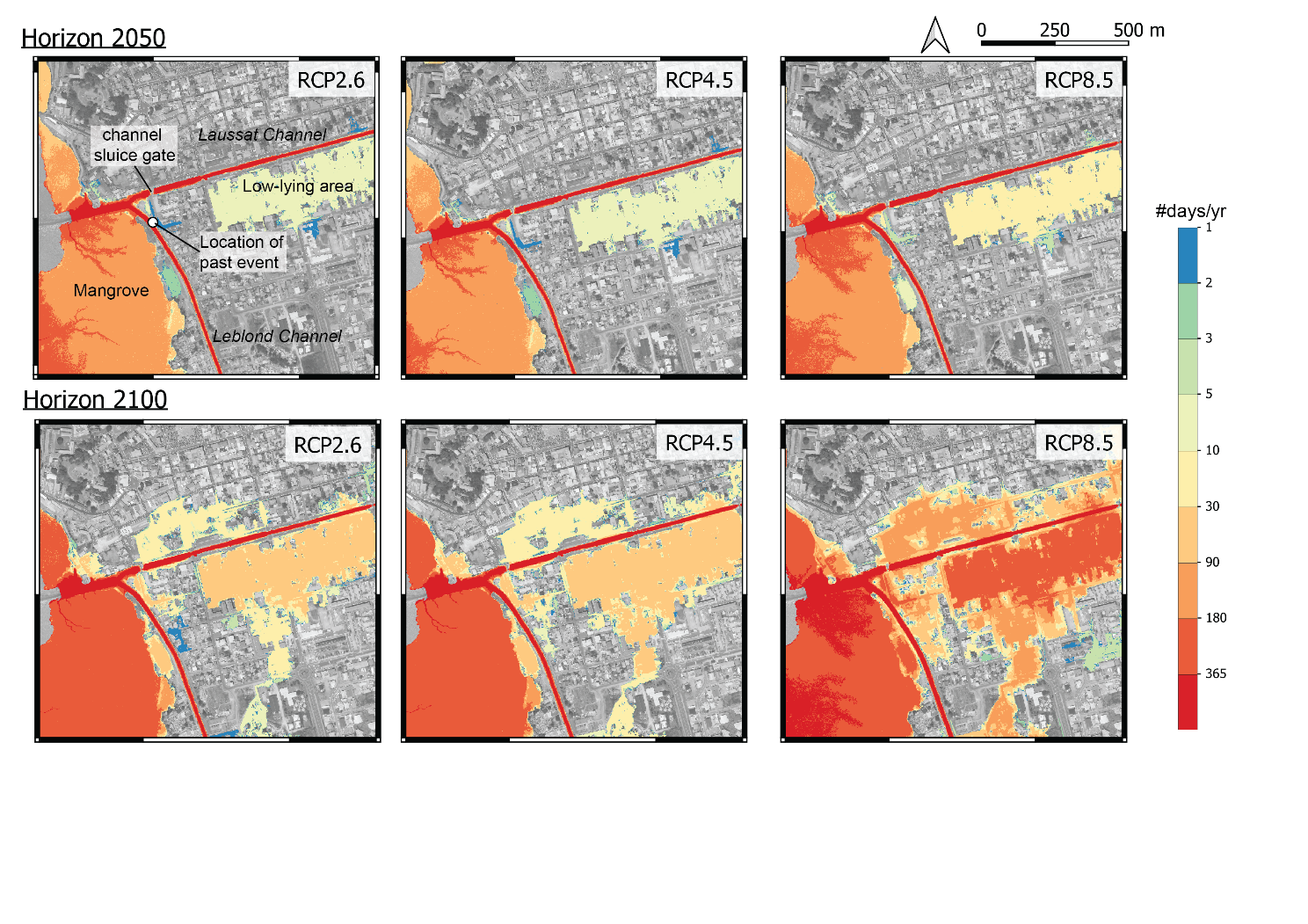


**SI Figure 8**. Projection of low-lying areas exposed to high-tide flooding (expressed in #days/year) in 2050 and 2100 for the RCP2.6, RCP4.5 and RCP8.5 median scenarios in Cayenne’s “Village Chinois” district (North-West). The mapping algorithm includes hydraulic connections at the surface of the DEM.

**Supplementary Material 11 – Daily maximum water level distribution @Ile Royale**


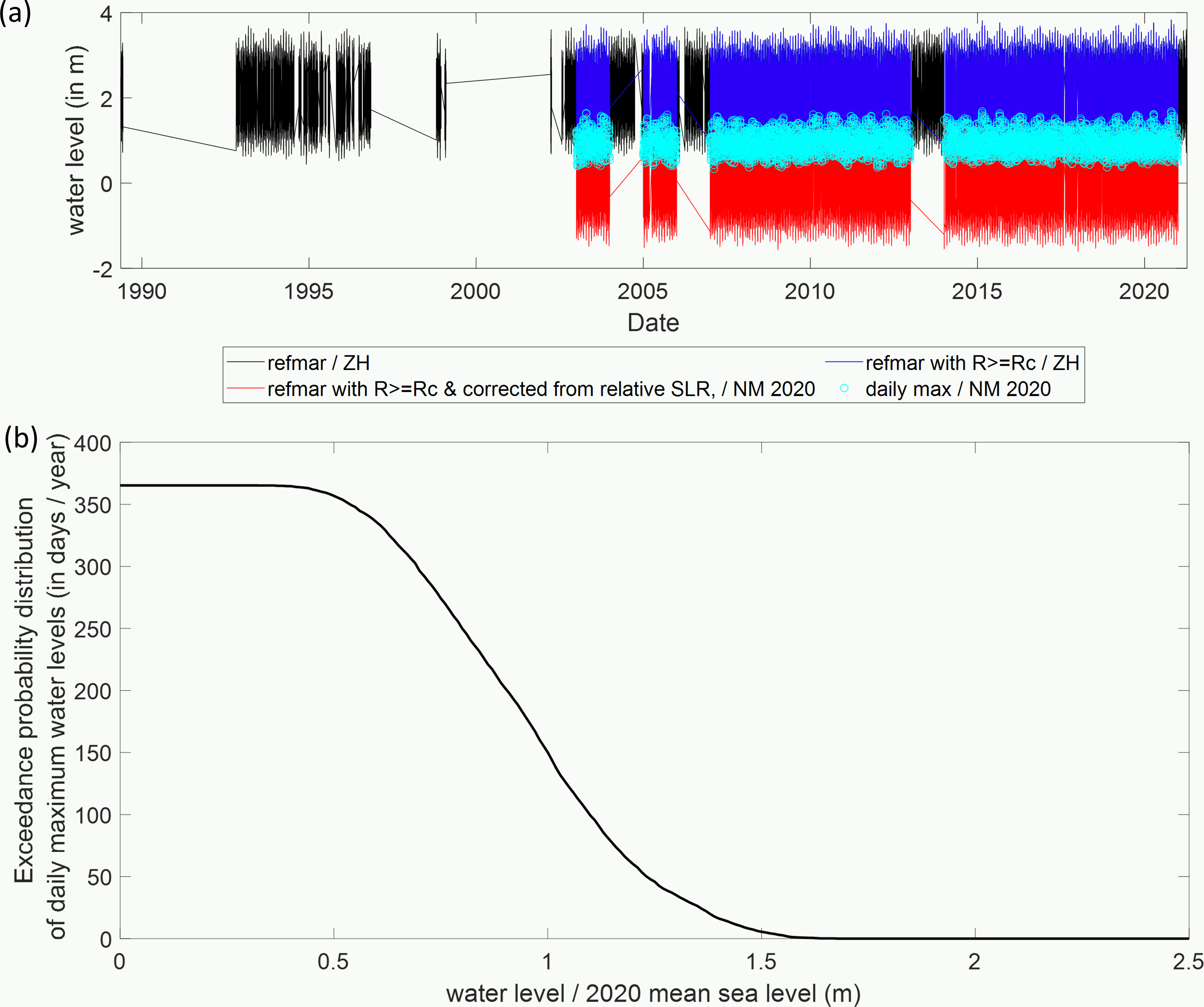


**SI Figure 9.** Processing of hourly tide gauge record at “Iles du Salut”. (a) water level timeseries at “Ile du Salut” resulting from the concatenation of the “Ile Royale – Anse Legoff” tide gauge (1989 to 2008) with the “Ile Royale” tide gauge (since 2006) with respect to the hydrographic zero (black). Only years with less than 10% of missing hourly data are selected (blue) and adjusted with respect to the mean sea level in 2020 (red). Daily maximum water levels are selected (cyan) to construct the (b) exceedance probability distribution (in days/year) of daily maximum water levels. Note that the local mean SLR linear trend (2.87 +/- 1.25 mm/year) has been removed. The difference between the observed hourly water level record and the predicted hourly water level due to tides (Supplementary Material 9) results in normally distributed residuals with a standard deviation of 6 cm and a maximum of ~20 cm (not shown).

**Supplementary Material 12 – Mapping low-lying areas exposed to chronic flooding**


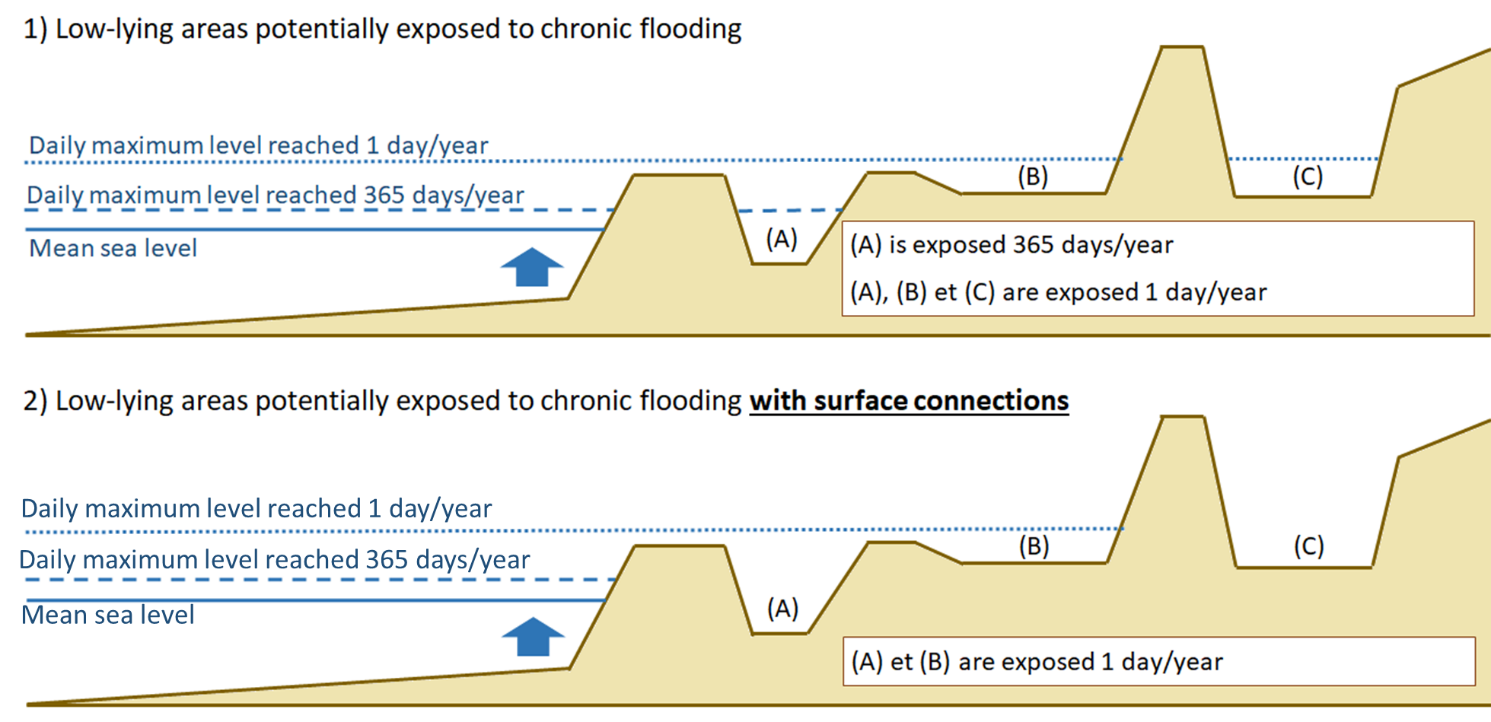


**SI Figure 10.** Schematic of the methods used to map low-lying areas potentially exposed to chronic flooding without (1) and with (2) DEM surface hydraulic connections accounted for.


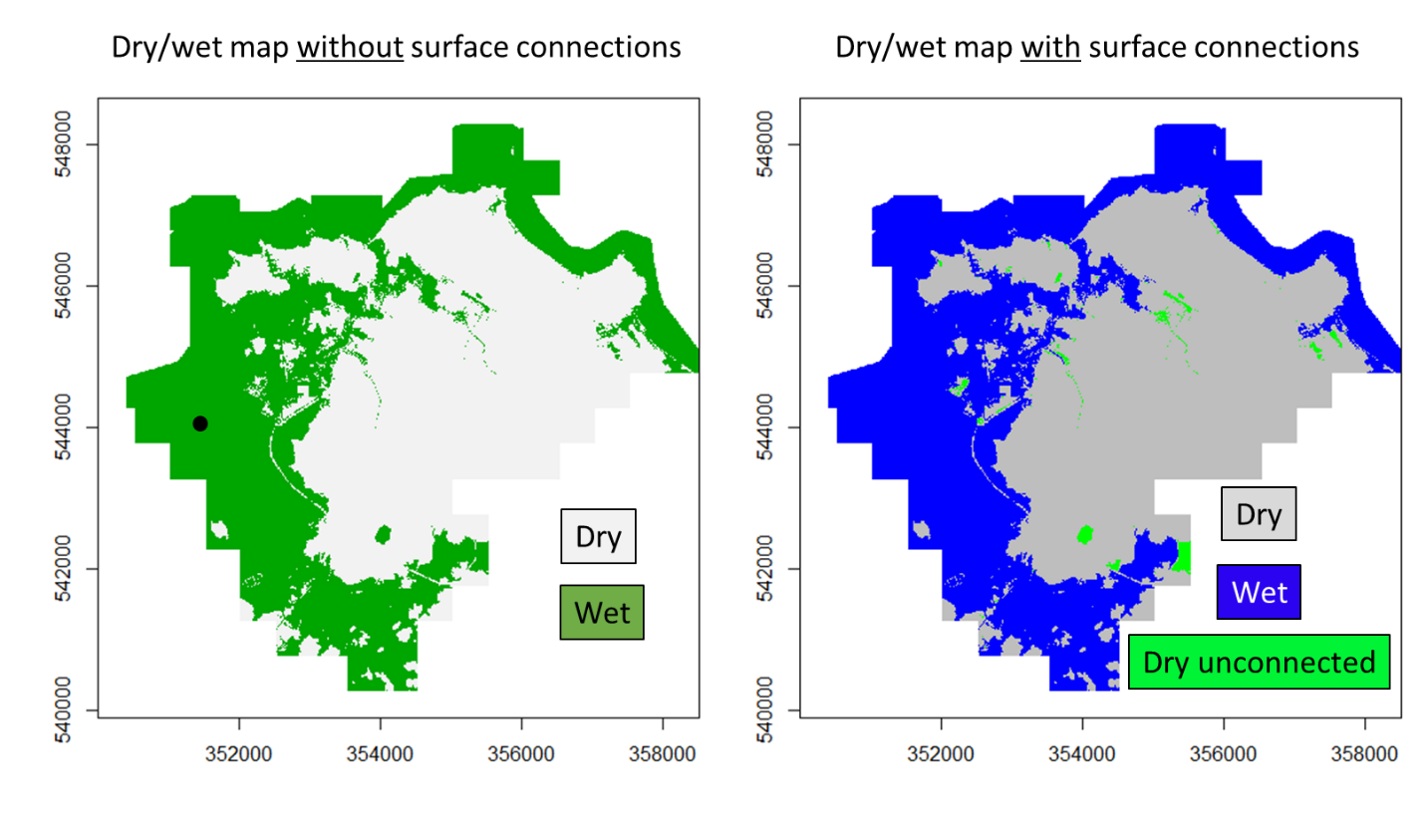


**SI Figure 11.** Example of low-lying area mapping under a 3m daily maximum level in Cayenne without (left) and with (right) DEM surface hydraulic connections accounted for. The areas in light green on the right are those that become dry after accounting for DEM surface hydraulic connections.

**Supplementary Material 13 - references**

Lyard, F. H., Allain, D. J., Cancet, M., Carrère, L., and Picot, N.: FES2014 global ocean tide atlas: design and performance, Ocean Sci., 17, 615–649, https://doi.org/10.5194/os-17-615-2021, 2021.
